# Comparing Prednisone and Methotrexate to Off-label Biologic Infliximab for Management of Ocular Uveitis: A Cost-minimization Analysis

**DOI:** 10.36469/9895

**Published:** 2015-01-20

**Authors:** William V. Padula, Miguel Cordero-Coma, Taygan Yilmaz, William V. Padula, Michéal J. Gallagher, Jonathan D. Campbell

**Affiliations:** 1 University of Chicago, IL, USA; 2 Hospital de Leon, Leon, Spain; 3 Harvard Vanguard Medical Associates, Boston, MA, USA; 4 Padula Institute of Vision, Guilford, CT, USA; 5 Hermitage Medical Clinic, Dublin, Ireland; 6 Center for Pharmaceutical Outcomes Research, University of Colorado, Aurora, CO, USA

**Keywords:** autoimmune disease, biologic, uveitis, ophthalmology, cost-minimization analysis

## Abstract

**Background:** Approximately 3.75% of cases of blindness in the United States are caused by uveitis. Incurred clinical costs and lost productivity related to vision loss in these cases totals $3.58 billion annually.

**Objective:** To evaluate whether infliximab, a modern off-label biologic, is cost-effective for treating posterior uveitis and panuveitis compared to current standards of care, methotrexate and prednisone.

**Methods:** A cost-effectiveness analysis using a Markov model to simulate a patient cohort with posterior uveitis or panuveitis. The model followed patients’ therapy from the onset of posterior uveitis or panuveitis using the U.S. societal perspective. The lifetime model simulated health states that could lead to successful reversal of uveitis with standard or intensified treatment with prednisone, methotrexate, or infliximab. Probabilities, health utilities, and costs were included in the model based on findings from the literature. We conducted univariate sensitivity analyses and a Bayesian multivariate probablistic sensitivity analysis to estimate uncertainty in results. Outcomes were measured in terms of costs ($US, 2010) and effects (qualityadjusted life years; QALYs) discounted at 3% per year were estimated for each simulated treatment. An incremental cost-effectiveness ratio (ICER) for pairwise results was interpretted assuming a predetermined willingness-to-pay threshold of $100,000/QALY.

**Results:** Average lifetime costs and QALYs for each drug were ($306.95; 15.80 QALYs) for prednisone, methotrexate ($36,232.24; 16.21 QALYs), and inflixmab ($74,762.63; 15.04 QALYs). Methotrexate was on average compared to prednisone, with an ICER of $86,901.16/QALY. Prednisone and methotrexate dominated infliximab. Sensitivity analyses suggested that the model was most sensitive to the utility for successful recovery from uveitis. The probabilistic sensitivity analysis returned results similar to the base case.

**Conclusion:** This cost-effectiveness analysis suggests that despite advances in the use of biologics for treating sight-threatening posterior uveitis and panuveitis, infliximab had lower effectiveness and higher costs compared to both prednisone and methotrexate. As compared to prednisone, methotrexate was associated with increased costs and QALYs and was found to be a good value. Clinical trials of infliximab in the uveitis population are needed to reduce the uncertain estimates of inflixmab treatment success and the drug’s cost-effectiveness.

## BACKGROUND

Non-infectious uveitides are comprised of a heterogeneous group of auto-immune inflammatory conditions accounting for about 10% of legal blindness and the fourth-leading cause of blindness worldwide.[1] Darrell et al. reported an incidence rate for uveitis of 17/100,000 person-years and a prevalence ratio of 204/100,000 over a 10-year period.[2] Up to 35% of patients with uveitis will develop cataracts, glaucoma, retinal detachment, cystoid macular oedema and other conditions that can lead to vision impairment or blindness.[3] According to Kirbach et al., uveitis accounts for approximately 10% of cases of blindness in the United States, which could represent a cost of as much as $3.58 billion annually in clinical costs and lost productivity related to vision loss.[4]

Based on the anatomical portion of the uvea involved, uveitis entities may be divided into four groups – anterior, intermediate, posterior, and panuveitis – which are characterized by different clinical signs, therapeutic management, and prognosis. Patients with posterior uveitis (i.e. intraocular inflammation primary involving the retina and/or choroid) or panuveitis (i.e. intraocular inflammation primary involving the anterior chamber, vitreous, and retina or choroid) represent about 37.5% of the total number of uveitis patients.[5] Although, the prevalence of posterior uveitis and panuveitis may differ throughout studies depending on geographic location and may be even higher in tertiary referral centres when compared to primary care settings. The fact that patients with posterior uveitis or panuveitis are at the greatest risk for adverse outcomes and vision loss is a noteworthy concern.

Traditional systemic therapeutic management of patients with posterior uveitis or panuveitis include the use of corticosteroids such as prednisone. Prednisone is considered in this study based on its widely recognized status as a mainstay in the treatment of uveitides.[6] Several immunosuppressor regimens have been explored in efforts aimed at induction of a durable remission without the associated risks of longterm corticosteroids. Methotrexate is a commonly used immunosuppressor for treating rheumatologic and ophthalmic conditions including posterior uveitis and panuveitis, which have shown refractory to or intolerant of systemic corticosteroids in a number of studies.[7] Methotrexate is presented in this study as the traditional corticoid-sparing therapy. However, in recent years the use of off-label biologic agents and particularly those inhibiting tumour necrosis factor alpha (TNF-a), a pro-inflammatory master cytokine, have been in widespread use among ophthalmologists worldwide.[8] Infliximab, a chimeric monoclonal antibody against TNF-a, has demonstrated successful results when used for treatment of non-infectious posterior uveitis and panuveitis patients who have been refractory or intolerant of traditional therapy.[9] These promising results support the increasing number of non-infectious posterior uveitis or panuveitis patients receiving infliximab.

According to Chu et al., despite the efficacy of biologics for treating ocular disease, the cost associated with a biologic infusion can exceed $1,400 per patient per month, which could affect payers' coverage choices.[10] Payments for off-label biologic prescriptions among uveitis patients are challenged by budgetary constraints and reorganizations to federal programs such as Medicare. For instance, the Centers for Medicare and Medicaid Services (CMS) only authorizes payment on another biologic, sipuleucel-T, for its approved indication and leaves determination off-label reimbursement to CMS regional contractors.[11] According to the American College of Rheumatology, cutting any form of reimbursement for biologics negatively impacts patient access, thereby limiting treatment options for rare diseases such as uveitis.[12] An observational study by Doshi et al. confirmed that infliximab utilization trends decreased significantly following the Medicare Modernization Act of 2003.[13] A cost-effectiveness analysis by Wailoo et al. demonstrated the value of covering infliximab in Medicare programs for rheumatoid arthritis, but evidence remains scarce on determining the value of infliximab for off-label usage.[14]

In the present study we evaluate whether infliximab, a modern off-label biologic anti-TNF-a agent is costminimzing as well as cost-effective for treating posterior uveitis or panuveitis patients when compared to traditional therapy including prednisone and methotrexate from the U.S. commercial payor perspective. An economic model compares the value of health interventions considering that the payment for all new treatments is not always possible as an aid to decision-making of preferable interventions. Multiple costeffectiveness analyses that evaluated biologics for treating related ophthalmic conditions including agerelated macular degeneration and diabetic macular oedema found biologics to be cost-effective from the payor perspective; although, these studies were financially supported by pharmaceutical companies.[15] This study fills a gap in financially independent research that evaluates the societal impact of biologics for ophthalmology. We hypothesize that based primarily on the excessive cost of infliximab; it will not serve as a cost-minimizing or cost-effective alternative to prednisone or methotrexate in the treatment of posterior uveitis.

## METHODS

### Study Design

This study implemented a Markov model comparing the cost and cost-utility of three treatment alternatives – prednisone, methotrexate, and infliximab – for patients diagnosed with posterior uveitis or panuveitis, based on guidelines set by the U.S. Panel on Cost-Effectiveness in Health and Medicine.[16] A Markov model of uveitis care is advantageous over a decision tree in economic analysis because it can capture transitionstates between treatment and endpoint conditions, including death and side effects associated with each pharmacotherapy. The cost-minimization analysis compared the direct costs of treatment and downstream outcomes. Cost-utility analysis can be interpreted by a ratio of incremental cost per quality-adjusted life year (QALY). Incremental costs represent the numerator of the ratio, and incremental effectiveness is in the denominator. All data for the model were extracted from previous literature indexed in MEDLINE. A rate of 3% per year was chosen for discounting costs and QALYs to represent the present values of future costs and outcomes.[17] All costs were corrected for inflation to represent 2010 $US.

This analysis was conducted from a U.S. commercial payor perspective since it measured all health effects and changes in resource consumption caused by the intervention that an insurance company would cover. This type of payor perspective includes the patient's full life expectancy beyond the period of treatment for posterior uveitis, assuming that the patient remains covered beyond a brief treatment window.

For patients who would undergo treatment for uveitis, the costs for this strategy were considered along with the probabilities in patient variation due to treatment intensity, vision-threatening uveitis, side effects, and death. All patients in the model were assumed to receive 1 year of therapy. After 1 year, those patients who did not have success from treatment for uveitis or experienced death transitioned into a mode of treatment failure such as low vision or side effects. The model cycled through a second year of treatment for those patients who endured the complication Cushing's syndrome as a side effect of prednisone. Probabilities and costs varied between treatment arms. Health utilities measured in QALYs reflected the transition-states of patients undergoing pharmacotherapy for uveitis as well as chronic condition endpoints.

**Figure 1.** Simplified View of the Semi-Markov Health Transition Process for Posterior Uveitis and Panuveitis

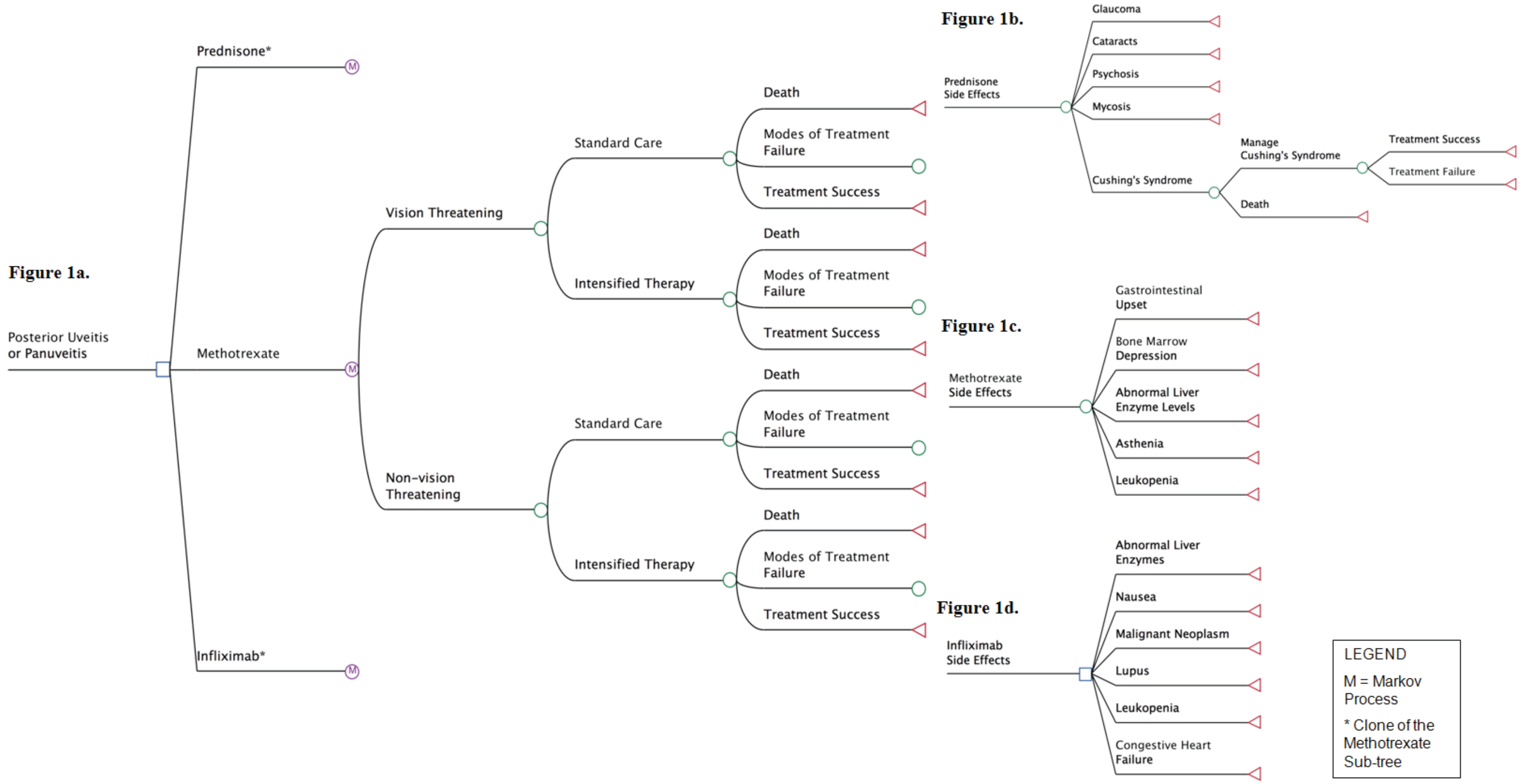

The model consists of 1-year cycles for a 2-year period of uveitis care. 1a.: Individuals receive prednisone, methotrexate or infliximab for uveitis treatment, and proceed through health states until reach treatment success, death, or certain side effect states. 1b., 1c., 1d.: Side effects are unique to each therapeutic and explained in the model description. While all health states are identical for each treatment arm, even probabilities, costs, and utilities differ between arms.

## Model

The 2-year model compared the cost-effectiveness of prednisone, methotrexate, and infliximab in the treatment of uveitis (Figure 1) utilizing TreeAge Pro Suite (TreeAge Software Inc., Williamstown, MA, 2009). For prednisone therapy, standard care was assumed as 50mg oral, daily, and intensified therapy was assumed as a maximum allowable dosage of 60mg daily.[18] Methotrexate oral therapy ranged from 10mg (standard care) to 15mg (intensified therapy) weekly.[19] Infliximab 4-week infusions ranged from 10mg/kg (standard care) to 16mg/kg (intensified therapy) for an average 50kg patient.[9] Following diagnosis of posterior uveitis or panuveitis, patients enter a treatment arm and are evaluated for 'vision threatening' or 'non-vision threatening' uveitis. The classification of uveitis results in varying probabilities of standard care or intensified therapy; generally, patients with non-vision threatening uveitis are more stable and likely to receive intensified therapy. Patients undergo a 12-month treatment regimen, after which they are monitored for treatment success, death, or modes of failure.

Patients who reach treatment success or death in the first year exit the study without any further incurred costs. For patients who enter a mode of failure, there are two alternative outcomes. First, a patient appears to gain no effect from taking the therapy and uveitis progresses to low vision. Second, certain side effects related to pharmacotherapy result in the discontinuation of treatments for posterior uveitis since the side effects are either more detrimental to the patient's health than uveitis (e.g. psychosis or asthenia), or the side effect makes the repair of uveitis irrelevant (e.g. cataracts or glaucoma). Third, Cushing's syndrome could develop as a side effect of prednisone while continuing to manage uveitis in year two. Management of Cushing's syndrome extends the patient's treatment for posterior uveitis into a second year, as long as death or Cushing's syndrome management failure does not occur first.

The side effects vary by each treatment arm. For prednisone, patients can experience end-state complications including mycosis, psychosis, glaucoma, cataracts, or Cushing's syndrome.[20] Patients on methotrexate are at risk for a number of end-state complications including asthenia, bone marrow depression, gastrointestinal upset, leukopenia, or abnormal liver enzyme levels.[21] Infliximab patients are at-risk for multiple end-state complications including congestive heart failure, nausea, lupus, malignant neoplasm, leukopenia, or liver enzyme complications.[22] Leukopenia is a predecessor to systemic infection and common to methotrexate and infliximab.

## Parameters of the Posterior Uveitis and Panuveitis Markov Care Model

The cost of each pharmacotherapy was calculated by a microcosting approach since treatment for uveitis consists of several factors (Table 1a). From the payor perspective there is cost of the drug, physician visit cost, patient follow-up visits, drug dispensation, and cost to manage any side effects during uveitis treatment. Drug cost was calculated as the average wholesale price (AWP, $2010 US) per dose. We assumed that the number of patient follow-up visits were equal between each arm of the study, and therefore negated; this assumption favours infliximab, which would likely have closer physician follow-up than methotrexate or prednisone as an off-label drug. Patients taking infliximab were allocated a drug dispensation cost for 13 annual infusions. An ophthalmologist visit cost reflecting the Medicare reimbursement rate (DRG 046) was added to all patients who experienced a mode of treatment failure since it was assumed that these patients would require a prognosis of failure or diagnosis of side effect.[23] Transition costs from standard care to intensified therapy are also shown in Table 1a. In the case of patients who developed manageable Cushing's syndrome, a second year of prednisone standard care cost was discounted at 3%.[24]

**Table 1a.** Parameters of the Posterior uveitis and panuveitis Markov Care Model: Annual Resource Costs ($US, 2010 values)

| Cost Component | Classification | Base Case | Range for Sensitivity Analysis | | | Description | Source |
|---|---|---|---|---|---|---|---|
| | | Cost ($) | Lower Bound | - | Upper Bound | | |
| Physician Visit | Follow-up Clinic visit* | 853.13 | 725.16 | - | 981.10 | Societal impact cost for patient treatment of uveitis | 23,32 |
| Drug Dispensation | Biologic Infusion | 139.32 | | | | Cost to provide patient with one infusion for infliximab | 34,35 |
| Prednisone | Pharmacotherapy | 7.24 | | | | AWP of 30-day prescription for 50mg oral, daily | 18,44 |
| Prednisone | Pharmacotherapy | 8.69 | | | | AWP of 30-day prescription for 60mg oral, daily | 18,44 |
| Standard Care | Annual Cost* | 86.88 | 73.85 | - | 99.91 | 12-month prescription of 50mg oral, daily | |
| Intensified Therapy | Annual Cost | 104.30 | | | | 12-month prescription of 60mg oral, daily | |
| | Transition Cost* | 17.42 | 14.81 | - | 20.03 | Cost difference between standard care and intensified therapy | |
| Cushing's Syndrome | Disease Management* | 26 755.59 | 22 742.25 | - | 30 768.93 | Annual disease management for Cushing's Syndrome | 24 |
| Methotrexate | Pharmacotherapy | 496.62 | | | | AWP of weekly prescription for 10mg oral, weekly | 33,44 |
| | Pharmacotherapy | 744.93 | | | | AWP of weekly prescription for 15mg oral, weekly | 33,44 |
| Standard Care | Annual Cost* | 25 824.24 | 21 950.60 | - | 29 698.28 | 52-week prescription of 10mg oral, weekly | |
| Intensified Therapy | Annual Cost | 38 763.36 | | | | 52-week prescription of 15mg oral, weekly | |
| | Transition Cost* | 12 939.12 | 10 998.25 | - | 14 879.99 | | |
| Infliximab | Pharmacotherapy | 3762.85 | | | | AWP of 10mg/kg (~500mg) 4-week infusion | 9,35,44 |
| | | | | | | | |
| | Pharmacotherapy | 6020.56 | | | | AWP of 16mg/kg (~800mg) 4-week infusions | 9,35,44 |
| | | | | | | | |
| Standard Care | Annual Cost* | 50 728.20 | 43 118.97 | - | 58 337.43 | 13 infusions of 500mg dose | |
| Intensified Therapy | Annual Cost | 80 078.40 | | | | 13 infusions of 800mg dose | |
| | Transition Cost* | 29 350.20 | 24 947.67 | - | 33 752.73 | 13 infusions of 800mg dose | |

* Cost classification used as input to the model; AWP, Average Whole Price ($US, 2010)

Probabilities were identified for each transitioning health state (Table 1b). A patient could only start out with posterior uveitis or panuveitis and transition to other comorbid states as a result of treatment failure. Treatment failure was divided into the probability of low vision or drug side effects. Death was probable at each node of the model. Most side effects resulted in no further probability of treatment success to relieve symptoms of uveitis. Only Cushing's syndrome offered some probability of treatment success.

For QALYs were used in the cost-utility analysis (Table 1c). All patients began with the same estimated utility of posterior uveitis (0.828 QALYs), which reflects the mean index score of a patient diagnosed with a disorder of the globe (ICD-9 360).[25] Patients who undergo standard care maintain this utility. Those who enter intensified therapy have a disutility of -0.006 QALYs, which is the disutility for the lowest 25th percentile for a diagnosis of disorder of the globe assuming that these patients endure greater potential drug-related harm with intensified therapy.[25]

Treatment success raises health utility to that of an average American age 50-64 (0.84 QALYs).[26] Patients who have a treatment failure, death or uncontrolled management of side effects have a reduced utility. Patients who die are assumed to have a utility of 0.0 QALYs. Patients with other side effects have the combined utility for uveitis in addition to a disutility for the specific diagnosis. In the case of Cushing's syndrome, the patient's final utility depends on whether or not uveitis symptoms are reversed.

Health utilities were discounted at 3% for remaining life expectancy. We assumed an average patient age of 51 at start of the study since this age reflected the mean of a diagnosed respondent in the Medical Expenditure Panel Survey (MEPS).[25] Lifetime health utility was then calculated for a remaining life expectancy of 27 years based on survival data from the Centers for Disease Control and Prevention (CDC).[27]

**Sensitivity Analysis**

One-way univariate sensitivity analyses were conducted to examine the impact that assumptions had on the model. The univariate sensitivity analyses were conducted by varying the base case estimates ±15% and assessing the impact on the model results (ranges appear in Table 2). The assumption for 15% variation was based on the fact that most parameters did not have evidence-based distributions when published in the literature. Tornado diagrams of all parameters were used to identify parameters with the highest relative impact on model results.

A Bayesian multivariate probabilistic sensitivity analysis (PSA) was done using 10,000 Monte Carlo simulations for all three therapeutic arms. The PSA applied distributions for each variable to characterize the impact of uncertainty on all parameters simultaneously to reflect a more realistic, heterogeneous patient population. Beta distributions were used for parameters with values ranging between 0.0 and 1.0 (i.e. probabilities and transition utilities), and gamma distributions were used for positive values >1.0 (i.e. costs and final health utilities).

**Table 1b.** Probabilities and Transition Probabilities

| Parameter | Base Case | Range for Sensitivity Analyses | | | Source |
|---|---|---|---|---|---|
| | Probability | Lower Bound | - | Upper Bound | |
| **Uveitis Type** | | | | | |
| *Vision Threatening* | 0.514 | 0.437 | - | 0.591 | 21 |
| Standard Care | 0.300 | 0.255 | - | 0.345 | 42 |
| Intensified Therapy* | 0.700 | N/A | - | N/A | |
| *Non-Vision Threatening** | 0.486 | N/A | - | N/A | |
| Standard Care | 0.092 | 0.0784 | - | 0.106 21 | |
| Intensified Therapy* | 0.908 | N/A | - | N/A | |
| **Prednisone** | | | | | |
| *Standard Care* | | | | | |
| Treatment Success | 0.740 | 0.629 | - | 0.851 | 43 |
| Death | 0.010 | 0.00850 | - | 0.0115 | 18 |
| Treatment Failure* | 0.250 | N/A | - | N/A | |
| Low Vision* | 0.748 | N/A | - | N/A | |
| Prednisone Side Effects | 0.252 | 0.214 | - | 0.290 14 | |
| *Intensified Therapy* | | | | | |
| Treatment Success | 0.820 | 0.697 | - | 0.943 | 43 |
| Death | 0.010 | 0.00850 | - | 0.0115 | 18 |
| Treatment Failure* | 0.170 | N/A | - | N/A | |
| Low Vision* | 0.601 | N/A | - | N/A | |
| Prednisone Side Effects | 0.399 | 0.339 | - | 0.459 | 14 |
| *Prednisone Side Effects* | | | | | |
| Glaucoma | 0.107 | 0.0901 | - | 0.123 | 20 |
| Cataracts | 0.107 | 0.0901 | - | 0.123 | 20 |
| Psychosis | 0.500 | 0.425 | - | 0.575 | 20 |
| Mycosis | 0.179 | 0.152 | - | 0.205 | 20 |
| Cushing's Syndrome* | 0.107 | N/A | - | N/A | |
| Death | 0.745 | 0.633 | - | 0.856 | 39 |
| Manage Cushing's Syndrome* | 0.255 | N/A | - | N/A | |
| Treatment Success | 0.541 | 0.460 | - | 0.622 | 36 |
| Treatment Failure* | 0.459 | N/A | - | N/A | |
| **Methotrexate** | | | | | |
| *Standard Care* | | | | | |
| Treatment Success | 0.900 | 0.765 | - | 0.950† | 40 |
| Death | 0.010 | 0.00850 | - | 0.0115 27 | |
| Treatment Failure* | 0.090 | N/A | - | N/A | |
| Low Vision* | 0.890 | N/A | - | N/A | |
| Methotrexate Side Effects | 0.110 | 0.091 | - | 0.123 | 40 |

*The complementary probability to other probabilities in the sub-tree

† An upper-limit on probability of treatment success with methotrexate since sub-tree probabilities cannot exceed 1.0

**Table 1b.** Probabilities and Transition Probabilities - Continued

| Parameter | Base Case | Range for Sensitivity Analyses | | | Source |
|---|---|---|---|---|---|
| | **Probability** | **Lower Bound** | **-** | **Upper Bound** | |
| **Methotrexate** | | | | | |
| *Intensified Therapy* | | | | | |
| Treatment Success | 0.950 | 0.808 | - | 0.980† | 40 |
| Death | 0.010 | 0.00850 | - | 0.0115 | 27 |
| Treatment Failure* | 0.040 | N/A | - | N/A | |
| Low Vision* | 0.890 | N/A | - | N/A | |
| Methotrexate Side Effects | 0.110 | 0.091 | - | 0.123 | 40 |
| *Methotrexate Side Effects* | | | | | |
| Gastrointestinal Upset | 0.273 | 0.232 | - | 0.314 | 21 |
| Bone Marrow Depression | 0.273 | 0.232 | - | 0.314 | 21 |
| Abnormal Liver Enzyme Levels | 0.182 | 0.154 | - | 0.209 | 21 |
| Asthenia | 0.021 | 0.0179 | - | 0.0241 | 21 |
| Leukopenia* | 0.251 | N/A | - | N/A | 21 |
| **Infliximab** | | | | 21 | |
| *Standard Care* | | | | | |
| Treatment Success* | 0.570 | N/A | - | N/A | |
| Death | 0.010 | 0.00850 | - | 0.0155 | 27 |
| Treatment Failure | 0.420 | 0.357 | - | 0.483 9 | |
| Low Vision* | 0.580 | N/A | - | N/A | |
| Infliximab Side Effects | 0.420 | 0.357 | - | 0.483 | 41 |
| *Intensified Therapy* | | | | | |
| Treatment Success* | 0.330 | N/A | - | N/A | |
| Death | 0.010 | 0.00850 | - | 0.0155 | 27 |
| Treatment Failure | 0.660 | 0.561 | - | 0.759 | 9 |
| Low Vision* | 0.230 | N/A | - | N/A | |
| Infliximab Side Effects | 0.770 | 0.655 | - | 0.886 | 41 |
| *Infliximab Side Effects* | | | | | |
| Abnormal Liver Enzymes | 0.130 | 0.110 | - | 0.150 | 42 |
| Nausea | 0.273 | 0.232 | - | 0.314 | 42 |
| Malignant Neoplasm | 0.013 | 0.0110 | - | 0.015 | 43 |
| Lupus | 0.065 | 0.0553 | - | 0.0748 | 42 |
| Leukopenia | 0.468 | 0.399 | - | 0.538 | 42 |
| Congestive Heart Failure* | 0.051 | N/A | - | N/A | 22 |

*The complementary probability to other probabilities in the sub-tree

† An upper-limit on probability of treatment success with methotrexate since sub-tree probabilities cannot exceed 1.0

**Table 1c.** Health Utilities and Final Rewards

| Parameter | Base Case | Range for Sensitivity Analyses | | | Source |
|---|---|---|---|---|---|
| | QALYs (Final Reward) | Lower Bound | - | Upper Bound | |
| **Year 1 QALYs** | | | | | |
| Standard Care with Uveitis | 0.828 | 0.704 | - | 0.952 | 25 |
| Transition Utility for Intensified Therapy | -0.006 | -0.00736 | - | -0.00544 | 25 |
| **Years 2-through-Life QALYs** | | | | | |
| Treatment Success | 0.840 (15.697) | 13.342 | - | 18.0515 | 26 |
| Treatment Failure: Low Vision | 0.694 (12.849) | 10.922 | - | 14.777 | 25 |
| Death | 0.0 (0.0) | 0.0 | - | 0.0 | Anchor |
| Abnormal Liver Enzyme Levels | 0.724 (13.410) | 11.399 | - | 15.422 | 25,26 |
| Asthenia | 0.793 (13.354) | 11.181 | - | 15.127 | 25 |
| Bone Marrow Depression | 0.764 (14.158) | 12.034 | - | 16.281 | 25 |
| Cataracts | 0.770 (14.270) | 12.129 | - | 16.410 | 25 |
| Congestive Heart Failure | 0.636 (11.766) | 10.001 | - | 14.777 | 25 |
| Cushing's Syndrome (Treatment Success) | 0.804 (14.715) | 12.507 | - | 16.922 | 25 |
| Cushing's Syndrome (Treatment Failure) | 0.716 (13.388) | 11.380 | - | 15.395 | 25,26 |
| Gastrointestinal Upset | 0.765 (14.176) | 12.050 | - | 16.303 | 25 |
| Glaucoma | 0.782 (14.494) | 12.320 | - | 16.668 | 25 |
| Leukopenia | 0.749 (13.877) | 11.796 | - | 15.959 | 25 |
| Lupus | 0.742 (13.746) | 11.685 | - | 15.808 | 26 |
| Mycosis | 0.738 (13.791) | 11.722 | - | 15.860 | 26 |
| Malignant Neoplasm | 0.847 (15.709) | 13.352 | - | 18.065 | 26 |
| Psychosis | 0.693 (12.831) | 10.906 | - | 14.755 | 25 |

QALY: Quality-adjusted life year

## RESULTS

### Expected Cost and Cost-utility

Prednisone was most affordable, followed by methotrexate. Infliximab, for its increased price, does not appear to reduce overall medical costs given a patient's likelihood of developing additional complications associated with costly care downstream. Based cost-minimization, infliximab is not a recommended alternative to the indicated standards of care.

According to the cost-utility analysis, methotrexate was cost-effective at a willingness-to-pay (WTP) threshold of \$100,000/QALY in 2010 \$US relative to prednisone, and infliximab was dominated (Table 2). The cost of prednisone was lowest at \$306.95 and had an effectiveness of 15.80 QALYs. Methotrexate cost \$36,232.24 and had the highest effectiveness of 16.21 QALYs. The incremental cost-effectiveness ratio (ICER) comparing methotrexate to prednisone was \$86,901.16/QALY. Infliximab cost the most at \$74,762.63 and its effectiveness was lower than prednisone at 15.04 QALYs, and therefore dominated by both methotrexate and prednisone.

**Table 2.** Discounted Expected Results of the Cost-effectiveness Analysis Comparing Methotrexate and Infliximab to Prednisone at Baseline for Treating Posterior Uveitis and Panuveitis

| Treatment | Cost ($) | Incremental Cost | Effect (QALY) | Incremental Effect | ICER ($/QALY) |
|---|---|---|---|---|---|
| **Prednisone** | 306.95 | | 15.80 | | |
| **Methotrexate** | 36 232.24 | 35 925.29 | 16.21 | 0.41 | 86 901.16 |
| **Infliximab** | 74 762.63 | 74 455.68 | 15.04 | -0.76 | (Dominated) |

QALY: Quality-adjusted life year; ICER: Incremental Cost-effectiveness Ratio

### Univariate Sensitivity Analyses

We performed univariate sensitivity analyses for each variable and stratified high-impact variables using a tornado diagram. The ±15% variation around the value of all cost, probability and utility estimates had some effects on the model results. Eleven different cost, probability and health utility parameters had an impact on the model results. Most of these parameters had values within the pre-specified variable range where the results might change so that methotrexate is no longer a cost-effective alternative to prednisone. There were no parameters that changed the results of the cost-minimization analysis. For the cost-utility analysis, the utility of treatment success, utility of treatment failure (i.e. low vision), and the utility of uveitis had the highest overall impact on the model results. Utilities of treatment success and treatment failure, as well as the utility of psychosis have values within the pre-specified range that affected the results of the model to sometimes favour prednisone over methotrexate.

The four probabilities for treatment success with standard care or intensified therapy regimens using methotrexate and prednisone impacted the model results within the pre-specified range as well. The probability of death also impacted the model results, but did not change the results within the 15% range. Additionally, the cost associated with standard care of methotrexate impacted the model results, but did not change the results within the pre-specified range.

Given the inherent design of the cost-utility analysis, which lacked an exact value for treatment success of infliximab, a 3-way univariate sensitivity analysis was also performed. This analysis pitched the probability of treatment success with methotrexate compared to the probabilities of death and infliximab side effects (i.e. the assumed complements of infliximab treatment success). The probabilities of each outcome ranged from 0.001-0.95, thereby maximizing the potential treatment effect of infliximab beyond a reasonable level of confidence. Despite this analysis, methotrexate was 100% cost-effective at the WTP threshold.

Overall, the univariate sensitivity analyses indicated that there is a fine line in supporting methotrexate as a cost-effective alternative to prednisone. Infliximab is not cost-effective in any scenarios. Given these considerations, there is some uncertainty as to whether methotrexate or prednisone is the conclusive choice at a WTP threshold of $100,000/QALY.

### Probabilistic Sensitivity Analysis

The PSA with 10,000 Monte Carlo simulations suggested that methotrexate was a cost-effective option at a WTP threshold of $100,000/QALY compared to prednisone. Infliximab was dominated by prednisone due to its negative ICER. An analysis of the net monetary benefits of each drug showed that below a WTP threshold of $93,000/QALY, methotrexate was not cost-effective. As such, prednisone dominated infliximab. The central tendencies of each drug's cost and effectiveness in the PSA reflected expected values of the base case results.

## DISCUSSION

This analysis suggests that prednisone and methotrexate exhibit good value and affordability relative to infliximab from the U.S. commercial payor perspective. Methotrexate is also cost-effective for the treatment of posterior uveitis and panuveitis according to findings of the cost-utility analysis. Despite advances in the use of biologics for treating this vision-threatening disease, infliximab may not offer a significant added value. Infliximab is dominated from a cost-utility standpoint in most PSA simulations as well compared to methotrexate, and prednisone is much less costly than either treatment at a lower effectiveness than methotrexate for a heterogeneous population.

Traditional corticoid-sparing therapy such as methotrexate should be considered for adoption for those uveitis patients refractory to or intolerant of systemic corticosteroids based on value. This conclusion remains in balance with expert opinion about what therapeutic agent is best for the patient presenting symptoms of uveitis.[28] The resulting patient-level costs of this study are similar to previously reported values from the cost burden analysis of uveitis done by Kirbach and colleagues.[4] In societies where the WTP threshold may be much lower than for this study, prednisone may be preferable to methotrexate for certain patient subgroups. It is important to note that prednisone is very affordable at a clinically meaningful level of effectiveness.

Existing concerns expressed by the American College of Rheumatology and other organizations regarding CMS reimbursement policies should not be withdrawn based solely on these results. The model for this analysis exhibits a number of side effects and potential adverse reactions for each drug. Patients presenting with uveitis who are withdrawn from methotrexate and prednisone for medical reasons are left with few other options before vision deteriorates further. Current patient cost-sharing programs through Medicare limit access of biologics to patients.[29] Clinical trials are needed to support or refute the use of infliximab in the uveitis population.

The study is limited by several factors. First, not all data are nationally representative. Some information are taken from randomized controlled trials of each drug, which reflect less generalizable evidence than postmarket observational studies on drug effectiveness.[30] Some of these clinical trials are for study populations that present certain ocular diseases that are not uveitis. Infliximab in particular has little evidence to support its efficacy – or lack thereof – for uveitis. As a result the model frames outcomes for patients treated with infliximab as the complements of other outcomes such as death. Additional clinical trials or observation studies need to be conducted to provide an improved reference of uveitis outcomes for infliximab. Second, the model does not cover all potential endpoints including forms of treatment failure, side effects and adverse events related to the use of these pharmacotherapies. Where literature lacked reliable information on parameter values for certain events, we chose to omit such states from the model and rely on the sensitivity analyses for capturing remaining uncertainty in the results. Third, treatment success and treatment failure for each therapeutic agent are not well-defined for uveitis.

Typically, patients who fail on one drug may switch to another, or undergo polypharmacotherapy. In this model drug groups are kept separate and the probability of treatment success or failure are the remaining proportion of patients after death and side effects. Thus, the probability of treatment success depends on each drug being efficacious for uveitis care and not resulting in failed cases that would lead an ophthalmologist to switch a patient's drug regimen. Fourth, distributions for the expected values of each parameter such as standard deviations or confidence intervals were not available for all parameters. The estimated 15% distributions represent a common approach in cost-effectiveness analysis that is less ideal than having published distributions, but necessary to effectively conduct sensitivity analyses for estimating uncertainty.

An additional limitation of this study is that health utilities in this model do not reflect the exact progression of posterior uveitis or panuveitis, and are not very sensitive to changes in health for multiple reasons. First, utilities are taken from a general diagnosis code for disorders of the globe. While this utility may closely represent that of a patient with uveitis, it is not an exact measure of health utility. Second, this study hypothesized the cost-effectiveness of a biologic for treating uveitis from the U.S. commercial payor perspective. The EQ5D index developed by Sullivan and Ghushchyan is the most thorough source of health utilities from nationally representative U.S. patient population through MEPS.[25] This index has population distributions for the diagnosis codes of uveitis and other disorders of the globe with knownfloor and ceiling effects, and less sensitivity than the Short-Form 6-Domain (SF6D) or Health Utility Index 3 (HUI3), which are commonplace in Canadian and European studies of heath utility. Because of these limitations, the conclusions of the cost-minimization analysis are emphasized over the cost-utility analysis, with hope that future iterations of this research can better quantify health utility of uveitis.

This is the first economic evaluation of its kind to evaluate the cost of treating uveitis with a variety of therapeutics that have become both standard care and experimental in ophthalmology. Based on these limitations, future economic research about therapeutics for uveitis would benefit from additional resources for health utility, or other effectiveness measures such as symptom free years, blindness avoided, or years of useful vision. A discrete trial or observational study on the use of infliximab, methotrexate and prednisone that includes these measures of its patient population would provide a useful reference for future work in this area and further refine the cost-effectiveness of uveitis treatment.

## CONCLUSION

In conclusion, the results of this economic analysis support the use of methotrexate to treat posterior uveitis and panuveitis. Prednisone is very affordable, and methotrexate is also less costly to treat uveitis than infliximab. Infliximab does not maintain as much value in this study; nonetheless, both could be clinically effective to certain patients. As Rosenfeld and Goodman pose, the question remains whether ophthalmologists should cross the line from standard care into off-label biologics for the treatment of uveitis in societies where it can be afforded.[31] Simulation studies such as this one can aid in adoption policy decision-making when randomized evidence is lacking on the head-to-head effectiveness of treatment alternatives. This model-based study suggests that more evidence is needed related to the treatment success (and failure) of infliximab and potentially other biologics. A trial designed to study infliximab as a secondline option for uveitis patients who have failed methotrexate or prednisone would be aligned with its current effectiveness and cost projections.

## CONFLICT OF INTEREST DECLARATION

The authors declare that they have no competing interests.

## References

[1] Nussenblatt RB: The natural history of uveitis. *Int Ophthalmol* 1990;14(5-6):303-8.

[2] Darrell RW, Wagener HP, Kurland LT. Epidemiology of uveitis: incidence and prevalence in a small urban community. *Arch Ophthalmol* 1962;68:502-14.

[3] Rothova A, Suttorp-van Schulten MS, Frits Treffers W, Kijlstra A: Causes and frequency of blindness in patients with intraocular inflammatory disease. *Br J Ophthalmol* 1996;80(4):332-6.

[4] Kirbach SE, Hayes OA, Cifaldi MA: The economic burden of uveitis. Annual Scientific Meeting; 2010 Nov; Atlanta, GA.

[5] Gritz DC, Wong IG: Incidence and prevalence of uveitis in Northern California; the Northern California Epidemiology of Uveitis Study. *Ophthalmology* 2004;111(3):491-500.

[6] Nussenblatt RB: Philosophy, goals and approaches to medical therapy. In: Nussenblatt RB, Whitcup SM, ed. Uveitis: Fundamentals and Clinical Practice. 4th ed. Philadelphia: Mosby; 2010.

[7] Kruh J, Foster CS: Corticosteroid-sparing agents: conventional systemic immunosuppressants. *Dev Ophthalmol* 2012;51:29-46.

[8] Sugita S, Kawazoe Y, Imai A, et al: Inhibition of the Th17 differentiation by anti-TNF-alpha therapy in uveitis patients with Behcet's disease. *Arthritis Res Ther* 2012;14(3):R99.

[9] Sukumaran S, Marzan K, Shaham B, Reiff A: High dose infliximab in the treatment of refractory uveitis: Does dose matter? *ISRN Rheumatology* 2012;2012:765380.

[10] Steinbrook R: The price of sight - Ranibizumab, bevacizumab, and the treatment of macular degeneration. *N Engl J Med.* 2006;355(14):1409-12.

[11] Chambers JD, Neumann PJ: Listening to Provenge - What costly cancer treatment says about future Medicare policy. *N Engl J Med.* 2011;364(18):1687-9.

[12] US House of Representatives: Ways and Means Committee, Subcommittee on Health. 2002. Medicare payments for currently covered prescription drugs: Statement of the American College of Rheumatology. http://waysandmeans.house.gov/Legacy/health/107cong/10-3-02/107-84final.htm.

[13] Doshi JA, Li P, Puig A: Impact of the Medicare Modernization Act of 2003 on utilization and spending for Medicare Part B-covered biologics in rheumatoid arthritis. *Arthritis Care Res* (Hoboken). 2010;62:352-60.

[14] Wailoo AJ, Bansback N, Brennan A, et al: Biologic drugs for rheumatoid arthritis in the medicare program: A cost-effectiveness analysis. *Arthritis Rheum* 2008;58:939-46.

[15] Mitchell P, Annemans L, Gallagher M, Hasan R, et al: Cost-effectiveness of ranibizumab in treatment of diabetic macular oedema (DME) causing visual impairment: evidence from the RESTORE trial. *Br J Ophthalmol* 2012;96:688-93.

[16] Luce B, Manning WG, Siegel JE, Lipscomb J: Estimating costs in cost-effectiveness analysis. In: Gold M, Siegel JE, Russell LB, Weinstein MC, ed. Cost-Effectiveness in Health and Medicine. New York: Oxford University Press; 1996:176-213.

[17] Lipscomb J, Weinstein MC, Torrance GW: Time Preference. In: Gold M, Siegel JE, Russell LB, Weinstein MC, ed. Cost-Effectiveness in Health and Medicine. New York: Oxford University Press; 1996:214-235.

[18] Multicenter Uveitis Steroid Treatment Trial Research Group, Kempen JH, Altaweel MM, Holbrook JT, et al: The multicenter uveitis steroid treatment (MUST) trial: Rationale, design, and baseline characteristics. *Am J Ophthalmol* 2010;149(4):550-61.e510.

[19] Sizto S, Bansback N, Feldman SR, Willian MK, Anis AH: Economic evaluation of systemic therapies for moderate to severe psoriasis. *Br J Dermatology* 2009;160:1264-72.

[20] Huscher D, Thiele K, Gromnica-Ihle E, et al: Dose-related patterns of glucocorticoid-induced side effects. *Ann Rheum Dis* 2009;68:1119-24.

[21] Gangaputra S, Newcomb CW, Liesegang TL, et al: Methotrexate for ocular inflammatory diseases. *Ophthalmology* 2009;116(11):2188-98.e2181.

[22] Suhler EB, Smith JR, Wertheim MS, et al: A prospective trial of infliximab therapy for refractory uveitis: preliminary safety and efficacy outcomes. *Arch Ophthalmol* 2005;123(7):903-12.

[23] Schappert SM, Rechtsteiner EA: Ambulatory medical care utilization estimates for 2006. In: U.S. Department of Health and Human Services, ed. Vol National Center for Health Statistics. Washington, DC: Centers for Disease Control; 2008 Aug.

[24] Swearingen B, Wu N, Chen SY, Pulgar S, Biller BMK. Health care resource use and costs among patients with cushing's disease. *Endocr Pract* 2011;17(5):681-90.

[25] Sullivan PW, Ghushchyan V: Preference-based EQ-5D index scores for chronic conditions in the United States. *Med Decis Making* 2006;26:410-20.

[26] Sullivan PW, Lawrence WF, Ghushchyan V: A national catalog of preference-based scores for chronic conditions in the United States. *Med Care* 2005;43:736-49.

[27] Xu J, Kochanek KD, Murphy SL, Tejada-Vera B: Deaths: Final Data for 2007. In: U.S. Department of Health and Human Services, ed. Vol 58. Atlanta, GA: Centers for Disease Control, National Vital Statistics Reports; 2010 May.

[28] Cochrane S, Dick AD: Tumor necrosis factor alpha-targeted therapies in uveitis. In: Pleyer U, Foster CS, ed. Uveitis and Immunological Disorders. Berlin: Springer; 2007:177-191.

[29] Liang SY, Haas JS, Phillips KA: Medicare formulary coverage for top-selling biologics. *Nature Biotechnol* 2009;27(12):1082-4.

[30] Rothwell PM: External validity of randomised controlled trials: "To whom to the results of this trial apply?". *Lancet* 2005;365:82-93.

[31] Rosenfeld PJ, Goodman KW: When is off-label drug use in the patient's best interest? *Am J Ophthalmol* 2009;147(5):761-63.

[32] Muennig P: MEDPAR Inpatient Hospital National Data for Fiscal Year 2004. Cost effectiveness analysis in health: A practical approach. Second ed. San Francisco: Jossey-Bass; 2008:203-48.

[33] Shah SS, Lowder CY, Schmitt MA, et al: Low-dose methotrexate therapy for ocular inflammatory disease. *Ophthalmology* 1992;99(9):1419-23.

[34] Thomson Healthcare: Red Book: Pharmacy's Fundamental Reference. In: PDR Editorial Staff, ed. 110th ed. New York: Thomson Healthcare; 2008.

[35] Vera-Llonch M, Massarotti E, Wolfe F, Shadick N, et al: Cost-effectiveness of abatacept in patients with moderately to severely active rheumatoid arthritis and inadequate response to methotrexate. *Rheumatology* 2008;47:535-41.

[36] Lobo A, Barton K, Minassian D, du Bois RM, Lightman S: Visual loss in sarcoid-related uveitis. *Clin Exper Ophthalmol* 2003;31:310-16.

[37] Cowan CL: Sarcoidosis. In: Yanoff M, Duker JS, ed. *Ophthalmology*. Vol 17. St. Louis, MO: Mosby; 1999:1-17.16.

[38] Thorne JE, Jabs DA, Qazi FA, et al: Mycophenolate mofetil therapy for inflammatory eye disease. *Ophthalmology* 2005;112:1472-7.

[39] Sherlock M, Ayuk J, Tomlinson JW, et al: Mortality in patients with pituitary disease. *Endocrine Rev* 2010;31:301-42.

[40] Dev S, McCallum RM, Jaffe GL: Methotrexate treatment for sarcoid-associated panuveities. *Ophthalmology* 1999;106:111-18.

[41] Nguyen QD, Hatef E, Kayen B, et al: A cross-sectional study of the current treatment patterns in non-infectious uveitis among specialists in the United States. *Ophthalmology* 2011;118(1):184-90.

[42] Tucker AL, Nembhard IM, Edmondson AC: Implementing new practices: An empirical study of organizational learning in hospital intensive care units. *Managment Science* 2007;53(6):894-907.

[43] Hanauer SB, Feagan BG, Lichtenstein GR, et al: Maintenance infliximab for Crohn's disease: The ACCENT I randomised trial. *Lancet* 2002;359(9317):1541-9.

[44] Thomson Healthcare. Red Book: Pharmacy's Fundamental Reference. In: PDR Editorial Staff, ed. 110th ed. New York: Thomson Healthcare; 2008.
